# Knowledge, Attitude and Perceptions Around Perinatal Mental Health Among Doctors in an Obstetrics and Gynaecology Academic Department in Singapore

**DOI:** 10.7759/cureus.38906

**Published:** 2023-05-11

**Authors:** Zi Xi Poo, Phai Ling Quah, Helen Chen, Ann Wright, Tiong Ghee Teoh, Lay Kok Tan, Kok Hian Tan

**Affiliations:** 1 Department of Maternal Fetal Medicine, Division of Obstetrics and Gynaecology, KK Women's and Children's Hospital, Singapore, SGP; 2 Department of Maternal Fetal Medicine, Division of Obstetrics and Gynaecology, KK Women’s and Children’s Hospital, Singapore, SGP; 3 Department of Psychological Medicine, KK Women’s and Children’s Hospital, Singapore, SGP

**Keywords:** asia, obstetrics and gynaecology, healthcare professionals, knowledge attitudes perception, perinatal mental health

## Abstract

Background

Frontline healthcare professionals who provide regular care to women in the antenatal and postnatal period play a critical role in the early detection and management of maternal perinatal mental health (PMH). This study aimed to assess the knowledge, attitudes, and perceptions of doctors around perinatal mental health in an obstetrics and gynaecology (O&G) department in Singapore.

Methods

Using an online survey, data was collected from 55 doctors who participated in the Doctor’s Knowledge, Attitudes and Perceptions of Perinatal Mental Health (I-DOC) study. The survey questions assessed the knowledge, attitudes, perceptions and practices in relation to PMH among doctors in the O&G specialty. Descriptive data was presented as means and standard deviations (SDs), or frequency and percentages.

Results

Out of the 55 doctors, more than half (60.0%) were not aware of the adverse impacts of poor PMH; 83.7% of doctors were not confident in providing PMH advice and 65.5% did not routinely screen patients for PMH disorders. There was a lower percentage of doctors (10.9% vs. 34.5%, p<0.001) who discussed PMH issues in the antenatal period compared to the postnatal period and this was statistically significant. Majority of doctors (98.2%) agreed that having standardised PMH guidelines will be useful. All doctors agreed on the benefits of having PMH guidelines, education and routine screening for patients.

Conclusion

There is inadequate PMH literacy among O&G doctors and lack of emphasis on antenatal PMH disorder. The findings highlighted the need for increased education and development of perinatal mental health guidelines.

## Introduction

Perinatal mental health (PMH) issues are prevalent in our society. They have a significant impact on women and their families and affect the physical, emotional and psychosocial development of their child [1]. It is important that doctors have the necessary knowledge, awareness, attitudes, and perceptions to recognize, diagnose, and treat PMH issues effectively. However, studies often suggest doctors lack sufficient knowledge and awareness of perinatal mental health issues or hold stigmatizing attitudes towards women with mental health issues. Bauer et al. published a study in 2017 showing that only 7% of doctors in the United Kingdom (UK) felt confident in their ability to diagnose perinatal depression, and 71% felt that they had not received adequate training in perinatal mental health to deal with mental health problems effectively. This highlighted the need for more education for healthcare professionals (HCPs) in this area [2].

While many aspects of maternal and child healthcare in Singapore have improved significantly, perinatal mental health problems such as depression and anxiety are still prevalent during pregnancy and the puerperium. A 2004 study by Chen et al. showed the prevalence of minor and major depression among antenatal women in the largest maternity unit in Singapore, KK Women’s and Children’s Hospital (KKH), was as high as 19.7% [3]. In a more recent prospective cohort study published in 2019, Lim et al. reported an antenatal depression rate of 9% [4]. A postnatal depression rate of 10.4% was found in another local cohort study ‘Growing Up in Singapore Towards Health Outcomes (GUSTO) [5].

There are several recognized adverse consequences of perinatal mood and anxiety disorders for both the mother and baby. These include marital discord, poor engagement with prenatal care, substance abuse, suicidal tendencies as well as impaired maternal-fetal bonding postnatally and high infanticide risks [1]. There are also obstetric risks of preterm birth, low birth weight and birth complications [4,5]. Maternal depression and anxiety have been found to impact fetal brain development structurally and functionally as well as have a long-term impact on cognitive developmental and behaviour [6-8]. The recent confidential enquiry into maternal deaths and morbidity between 2018 and 2020, Saving Lives, Improving Mothers’ Care 2022 showed that deaths by suicide had risen threefold since the previous report, with suicide being the leading direct cause of death during pregnancy, childbirth and the year after birth at the time of publication [9].

It is vital to raise awareness among frontline HCPs caring for pregnant women of signs and symptoms of anxiety and depression in their patients and generally improve knowledge of the negative impact of poor mental health on pregnancy. Early detection of PMH disorders allows early intervention that improves outcomes for both mother and baby.

The aim of this study was to assess the knowledge, attitudes, perceptions and practices among doctors in relation to perinatal mental health in an obstetrics and gynaecology (O&G) academic department in Singapore.

## Materials and methods

Study sample

A convenience sampling method was used to recruit participants from the KK Women’s and Children’s Hospital and Singapore General Hospital (SGH) for this survey. Eligible participants were doctors affiliated to the O&G department based in KKH and SGH. There are 110 O&G doctors in our SingHealth Institution that provided antenatal and postnatal care. To have a confidence level of 95% so that the real value was within ±10% of the surveyed value, we aimed for at least 52 responses. Participants were invited to answer an anonymous electronic online survey via FormSG (Government Technology Agency, Singapore). The survey was conducted over a four-month period between September 2022 and January 2023. Research procedures in this study were approved by the SingHealth Centralised Institutional Review Board.

Doctor's Knowledge, Attitudes and Perceptions of Perinatal Mental Health (I-DOC) survey

Data Collection

The survey comprised 13 items that collected data on (1) participant provider level, (2) awareness, (3) attitudes and (4) practices of perinatal mental health. The survey took an average of 10 minutes to complete.

The questions centered on the following domains.

Doctor’s Awareness of Perinatal Mental Health

Participants were assessed on their awareness of perinatal mental health, with two questions: (1) “Are you aware of adverse pregnancy outcomes, and child developmental outcomes related to perinatal depression or anxiety?” and (2) “Are you aware of any perinatal mental health guidelines for the Singapore population?”

Doctor’s Attitudes Towards Perinatal Mental Health

Participants were assessed on their attitudes toward perinatal mental health, with four questions: (1) “Do you think having standardised perinatal mental health guidelines will be useful for healthcare practitioners in Singapore to enable holistic patientcare?”, (2) “Do you think that having perinatal mental health guidelines will be useful for your patients who are pregnant or have recently delivered?”, (3) “Do you think that educating patients on mental health during pregnancy or post-pregnancy is important?” and (4) “Do you think screening for depression during pregnancy or post-pregnancy is beneficial for patients?”

Doctor’s Practices on Perinatal Mental Health

Participants were assessed on their practices on perinatal mental health with four questions: (1) “Do you ever recommend practicing healthy lifestyle habits (i.e., balanced diet, smoking cessation or alcohol abstinence, sufficient exercise and sleep) to patients during pregnancy or post-pregnancy for the benefit of their mental health?”, (2) “Do you routinely screen for depression in your antenatal or postnatal patients?”, (3) “If someone initiates a discussion about mental health with your patient during pregnancy or post-pregnancy, who will it usually be?” and (4) “How confident are you currently in providing advice on mental health to your patients during pregnancy or post-pregnancy?”

The participants were also asked if they initiated any discussion about mental health with their patients, and if their patients self-reported mental health separately during the antenatal and postnatal period using a five-point Likert scale response: “never”, “rarely”, “sometimes”, “often” and “always.”

Statistical analyses

Descriptive data were presented as frequencies and percentages. The comparison between doctor-initiated discussion about mental health and patient self-reported mental health between the antenatal and postnatal periods was assessed using the McNemar’s chi-square test. All analyses were performed using the Stata software, version 13 (StataCorp, College Station, TX).

## Results

Out of 140 O&G doctors in the SingHealth Institution, about 110 provided antenatal and postnatal care for patients. In our study, we obtained a total of 55 responses (50.0%) from these 110 O&G doctors. Of the 55 respondents, 19 (34.5%) were medical officers, 11 (20.0%) were senior residents, 5 (9.1%) were associate consultants, 11 (20.0%) were consultants, and 9 (16.4%) were senior consultants.

Doctor’s awareness, attitudes and practices on perinatal mental health

Among the 55 respondents, 33 (60.0%) were not aware of adverse pregnancy outcomes as well as poorer child developmental outcomes related to perinatal depression or anxiety compared to those who did (22; 40.0%). Most doctors (54; 98.2%) were not aware of any existing PMH guidelines in Singapore.

The majority (54; 98.2%) of doctors agreed that having standardized PMH guidelines would be beneficial for healthcare workers in Singapore to practice holistically when dealing with pregnant or recently delivered patients. All 55 doctors (100%) agreed that mental health education and routine screening during pregnancy or post-pregnancy is beneficial and important for patients (Table 1).

**Table 1:** Questions on awareness and attitudes of O&G doctors around perinatal mental health O&G: obstetrics and gynaecology

	Yes, n (%)	No, n (%)
Questions on awareness		
Are you aware of adverse pregnancy outcomes, and child developmental outcomes related to perinatal depression or anxiety?	22 (40.0)	55 (60.0)
Are you aware of any perinatal mental health guidelines for the Singapore population?	54 (98.2)	1 (1.8)
Questions on attitudes		
Do you think having standardised perinatal mental health guidelines will be useful for healthcare practitioners in Singapore to enable holistic patientcare?	54 (98.2)	1 (1.8)
Do you think that having perinatal mental health guidelines will be useful for your patients who are pregnant or have recently delivered?	55 (100)	0 (0)
Do you think that educating patients on mental health during pregnancy or post-pregnancy is important?	55 (100)	0 (0)
Do you think screening for depression during pregnancy or post-pregnancy is beneficial for patients?	55 (100)	0 (0)

Just under half (27; 49.1%) of the doctors said that they recommended a healthy lifestyle habit to women during pregnancy or post-pregnancy for the benefit of their mental health; 11 (20.0%) doctors reported that they would only make such recommendations if the discussion on mental health was initiated by the patient. The survey revealed that 26 (47.3%) doctors would initiate conversation about mental health issues rather than waiting for patients to report themselves (Table 2).

**Table 2:** Questions on practices of O&G doctors about perinatal mental health O&G: obstetrics and gynaecology

Questions on practices	n (%)
(1) Do you ever recommend practicing healthy lifestyle habits (i.e., balanced diet, smoking cessation or alcohol abstinence, sufficient exercise, and sleep) to patients during pregnancy or post-pregnancy for the benefit of their mental health?	
Never	2 (3.6)
Rarely	2 (3.6)
Sometimes	13 (23.6)
Mostly	16 (29.1)
Always	11 (20.0)
Only if the patient initiates a discussion	11 (20.0)
(2) Do you routinely screen for depression in your antenatal or postnatal patients?	
No	36 (65.5)
Yes	19 (34.5)
(3) If someone initiates a discussion about mental health with your patient during pregnancy or post-pregnancy, who will it usually be?	
This discussion is never initiated	2 (3.6)
I will be the one to initiate a discussion	26 (47.3)
My patients will initiate a discussion	25 (45.5)
My staff will initiate a discussion	2 (3.6)
(4) How confident are you currently in providing advice on mental health to your patients during pregnancy or post-pregnancy?	
Not confident	20 (36.4)
Somewhat confident	26 (47.3)
Confident	8 (14.5)
Very confident	1 (1.8)

The majority (46; 83.7%) of doctors were not fully confident [comprised somewhat confident (26; 47.3%) or not confident (20; 36.4%)] in providing mental health advice to patients; 36 (65.5%) doctors reported that they did not routinely screen patients for mental health issues. Out of the 19 (34.5%) doctors who replied “yes” to screening for mental health, six doctors (31.6% of 19) reported that no validated assessment tools were used.

Discussion on perinatal mental health in the antenatal and postnatal periods

**Figure 1:**
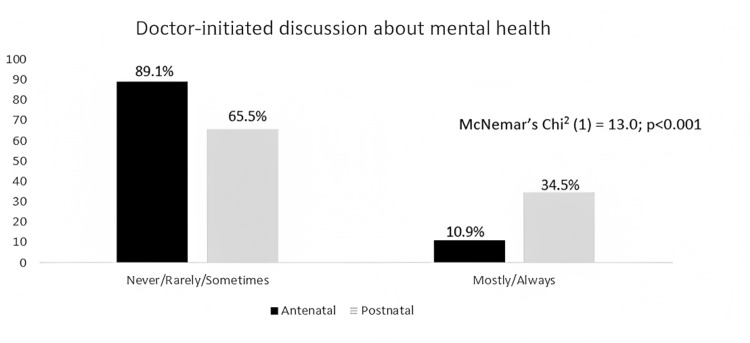
Percentages for doctor-initiated discussions of perinatal mental health issues

Figure 1 shows that the overall percentage of doctors who frequently (mostly or always) discussed mental health issues was lower than those who seldom initiate mental health discussion. Of those doctors who initiated mental health discussion, more tended to do so in the postnatal period (34.5%) compared to the antenatal period (10.9%), and this was statistically significant (p value 0.001).

**Figure 2:**
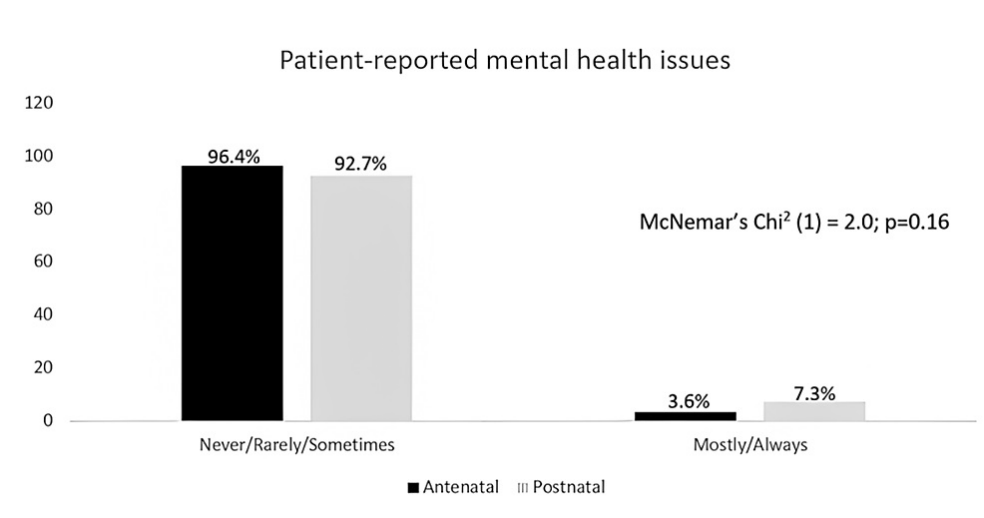
Percentages for patients' self-reported perinatal mental health issues during the antenatal and postnatal periods

Figure 2 shows patients' self-reported issues of mental health, for which similar trends were seen. A higher percentage of patients (96.4%) seldom reported these issues in the antenatal period, compared to the postnatal period (92.7%). There was also a lower percentage of patients who often self-reported mental health issues, and those who did so were more likely to do so in the postnatal period (7.3%) compared to the antenatal period (3.6%), p>0.05.

## Discussion

To the best of our knowledge, this is the first study specifically focusing on O&G doctors’ knowledge, awareness, attitudes and perceptions when it comes to PMH issues in Southeast Asia. Southeast Asian countries are diverse with respect to ethnicity, language, culture, and economic status with some falling into middle- and low-income groups. There is inequity of access to skilled mental healthcare and PMH problems are often under-recognized and undertreated.

The results of our study suggest a significant literacy gap in O&G doctors in Singapore regarding knowledge about how to detect perinatal mental health disorders or available guidelines and interventions available to treat them. This, in combination with an only partially integrated obstetric and mental health service, has led to a low level of willingness to address it with patients who, in turn, appear reticent in raising PMH issues without being prompted.

Comparison with other studies

There are limited papers published on doctor’s knowledge, attitudes and perceptions regarding PMH issues. A systematic review of perceptions and attitudes around PMH in Bangladesh, India and Pakistan by Insan et al. showed that healthcare professionals’ attitudes are still heavily influenced by societal pressures and stigma such that they avoid asking about emotional or mental health symptoms as they perceive it to be inappropriate [10]. In addition, they also found that many healthcare providers had poor awareness and understanding of PMH and did not perceive them to be serious health concerns [10]. This, also in part, stems from societal and cultural behaviours especially in the Asian context where women with mental health conditions are perceived to be ‘weak’ or ‘mad’ and risk having their child taken away from them [11]. Another review by Mannava et al. demonstrated that healthcare professionals' negative attitudes and behaviours can undermine healthcare-seeking behaviours and affect patient well-being and satisfaction [12].

Smith et al. published a systematic review and meta-analysis looking at the barriers to accessing mental health services for women with perinatal mental illness in the UK. They found several complex multilevel barriers to accessing mental health services, of which HCPs constitute the firstline barrier due to their lack of knowledge and perceived lack of training in PMH [13]. Furthermore, HCPs tended to dismiss low moods or anxiety as part of normal pregnancy symptoms. There was a general lack of knowledge among HCPs about care pathways, resulting in delayed referral to appropriate services [13]. Similarly, in Virginia, United States, Leiferman et al. surveyed 232 primary care physicians, which included obstetricians and family medicine practitioners, and found that although majority (90%) agreed it was their responsibility to recognize maternal depression, 40% rarely assessed for depression and 66% rarely provided a referral; there were significant differences in beliefs and practices among the physicians [14]. Another paper by Ali et al. looking at midwives’ perspectives on PMH in Pakistan similarly showed that midwives, being the primary care provider for most childbirth in Pakistan, lacked competencies related to PMH such as assessment and understanding of PMH, resulting in delay of management of PMH issues [15].

Limited resources such as lack of time during clinic consultations has also been raised as a key barrier to assessing patients’ mental health status and to provide adequate counselling [16]. The Royal College of Obstetricians and Gynaecologists (RCOG) Maternal Mental Health survey in 2017 reported that women often feel rushed during consultations, usually attributed to an overstretched service rather than doctors not caring and this limits their ability to voice their mental health problems [17].

Antenatal versus postnatal

The few doctors who did broach the subject of mental health issues mostly did so in the postnatal period and mainly focused on the subject of depression. In our study, approximately 90% of doctors and almost all patients rarely brought up issues of PMH during the antenatal period. The lack of emphasis on antenatal PMH disorder was evident from a similar study in the UK that reported that women with antenatal depressive symptoms were often ignored and were told by healthcare professionals to wait until after birth to seek treatment for their conditions [17]. The American College of Obstetricians and Gynecologists (ACOG) Committee reported that perinatal depression affects one in seven women, with a 27% incidence of pre-existing depression, 33% occurring antenatally and 40% occurring postpartum; hence, they recommended screening both antenatally and postnatally [18]. Talking about mental wellbeing especially in the antenatal period is also often overlooked in antenatal educational classes, other than a brief mention of baby blues and postnatal depression [17]. This oversight needs to be addressed, as antenatal anxiety and depression can be as significant as postnatal depression, and in some cases can lead to women choosing to terminate their pregnancy or even commit suicide rather than continue unsupported.

Use of validated tools and questionnaires

In our study, only a third of O&G doctors routinely screened their patients using validated assessments for PMH issues. Perceptions on the use and values of assessment tools were mixed. There is a lack of uniformity among psychometric tests used that makes acceptability towards them difficult to measure. Much of the PMH research is focused on the Edinburgh Postnatal Depression Scale (EPDS) as it is the most frequently used screening tool [19]. However, there has often been a lack of consensus on the ideal frequency of screening and onward referral completion rates. In the meta-analysis by Smith et al., HCPs often expressed negativity towards the use of existing assessment tools like EPDS as they felt these were just “tick-box exercises”; the unclear policy around the appropriate and acceptable use of assessment tools has caused women with PMH issues to be missed, resulting in delayed referral [13]. Another systematic review and meta-analysis by Fellmeth et al. looked at validated screening tools to identify common mental disorders in perinatal and postpartum women in India; it showed that EPDS had a pooled sensitivity of 88.9% and specificity of 93.4% in identifying perinatal depression; EPDS has therefore been deemed psychometrically valid even in diverse Indian settings and is recommended for routine usage to improve detection of perinatal depression [20].

Areas of improvement

Our study revealed that doctors lacked sufficient knowledge in standard screening and management of PMH disorders. Even for doctors who were open to assessing women’s mental health status, they often felt inhibited by the lack of formal training and knowledge to correctly identify, counsel and manage women with mental health issues [21]. Other studies have also shown that a lack of awareness and understanding of PMH issues among HCPs is one of the primary barriers to women accessing help. This has highlighted the need for standardized PMH guidelines as well as education. From our study, majority of doctors (98.2%) agreed that having PMH guidelines will be useful. All doctors uniformly agreed on the benefits of education and routine screening.

A key step in improvement is increasing awareness and training of doctors in the recognition and management of perinatal mental health problems [13]. This should help to break the cycle of stigma among patients and HCPs. This would also empower doctors to counsel their patients with confidence. Women would also be more receptive to report and discuss about their mental health. Counselling in a friendly, non-judgmental and confidential manner has been shown to remove stigma and shame associated with perinatal mental health issues and provides the reassurance of a psychological safe space to engage the healthcare professional. This is preferable to pharmacological drugs as firstline treatment [22].

In addition, inter-professional simulation training has been found to improve collaboration and awareness among HCPs and other key stakeholders like midwives and nurses, and lead to improvement in self-efficacy with skills, knowledge and confidence to manage the problems [23].

In recognition of the importance of PMH in maternal and child outcomes, more focus is now being placed locally on the medical, psychological, and counseling services in this area that should improve cohesion between obstetricians and PMH services and ease access for patients.

Limitations

The limitation of this study was a relatively small sample size. The response rate was 50.0%, out of the 110 doctors providing obstetric care. While it is a relatively good representation of local practices in the largest maternity unit in Singapore, a bigger sample size would be ideal.

## Conclusions

There is inadequate PMH literacy among O&G doctors and also lack of emphasis on antenatal PMH disorders. Our study findings indicate a need for perinatal mental health guidelines in Southeast Asia for healthcare professionals to increase their levels of knowledge, as well as confidence and skills in the detection and management of maternal perinatal mental health. This in turn should result in timely interventions for women with perinatal mental health issues and improve outcomes for the mother, child and family.
